# Multi-Tissue Transcriptomic-Informed In Silico Investigation of Drugs for the Treatment of Dengue Fever Disease

**DOI:** 10.3390/v13081540

**Published:** 2021-08-04

**Authors:** Beatriz Sierra, Ana Cristina Magalhães, Daniel Soares, Bruno Cavadas, Ana B. Perez, Mayling Alvarez, Eglis Aguirre, Claudia Bracho, Luisa Pereira, Maria G. Guzman

**Affiliations:** 1 Virology Department, PAHO/WHO Collaborating Center for the Study of Dengue and its Vector, Pedro Kourí Institute of Tropical Medicine (IPK), Havana 11400, Cuba; siebet@ipk.sld.cu (B.S.); anab@ipk.sld.cu (A.B.P.); mayling@ipk.sld.cu (M.A.); eglis@ipk.sld.cu (E.A.); cbracho@ipk.sld.cu (C.B.); lupe@ipk.sld.cu (M.G.G.); 2 i3S-Instituto de Investigação e Inovação em Saúde, Universidade do Porto, 4200-135 Porto, Portugal; acmagalhaes@ipatimup.pt (A.C.M.); danielcarpinteirosoares@gmail.com (D.S.); bcavadas@ipatimup.pt (B.C.); 3 IPATIMUP—Instituto de Patologia e Imunologia Molecular, Universidade do Porto, 4200-135 Porto, Portugal; 4 ICBAS—Instituto de Ciências Biomédicas Abel Salazar, Universidade do Porto, 4050-313 Porto, Portugal

**Keywords:** Dengue fever disease, multi-tissue transcriptomics, in silico evaluation of drugs, tissue specialization, common mechanisms of action

## Abstract

Transcriptomics, proteomics and pathogen-host interactomics data are being explored for the in silico–informed selection of drugs, prior to their functional evaluation. The effectiveness of this kind of strategy has been put to the test in the current COVID-19 pandemic, and it has been paying off, leading to a few drugs being rapidly repurposed as treatment against SARS-CoV-2 infection. Several neglected tropical diseases, for which treatment remains unavailable, would benefit from informed in silico investigations of drugs, as performed in this work for Dengue fever disease. We analyzed transcriptomic data in the key tissues of liver, spleen and blood profiles and verified that despite transcriptomic differences due to tissue specialization, the common mechanisms of action, “Adrenergic receptor antagonist”, “ATPase inhibitor”, “NF-kB pathway inhibitor” and “Serotonin receptor antagonist”, were identified as druggable (e.g., oxprenolol, digoxin, auranofin and palonosetron, respectively) to oppose the effects of severe Dengue infection in these tissues. These are good candidates for future functional evaluation and clinical trials.

## 1. Introduction

Dengue virus (DENV) is an arbovirus (Flaviviridae family) with four genetically distinguishable serotypes (DENV-1 to DENV-4), whose rapid worldwide spread is a major health problem [1]. The virus is transmitted to humans through the bite of infected *Aedes aegypti* or *Aedes albopictus* mosquitoes [2]. It is estimated that Dengue has an incidence of 390 million infections per year, of which 96 million people manifest the disease clinically and more than 20,000 die [3], contributing to a total annual global burden of 8.9 billion dollars [4]. Increases in Dengue global incidence and geographical distribution are expected due to the rise of global temperature, the increase of worldwide travel and the rapid growth of densely populated zones [5].

After being infected by the virus, patients can either be asymptomatic (~75% of cases) or, after a 4–10 day incubation period, develop symptoms that range from those of a mild flu to more severe symptoms [1]. The milder form of the disease (Dengue Fever—DF) is characterized by a rapid onset of fever accompanied by severe headaches, myalgias, arthralgias and gastrointestinal discomfort that last 7–14 days, after which homeostasis is normally restored. The more severe cases (Dengue Haemorrhagic Fever—DHF) present coagulopathy, spontaneous bleeding, low to moderate liver injury and increased vascular fragility and permeability. DHF can eventually progress to an even more severe case (Dengue Shock Syndrome—DSS), characterized by rapid fluid loss that leads to severe hypotension and haemorrhagic episodes, mainly bleeding in the skin and the gastrointestinal tract. DHF and DSS can sometimes lead to death. Several factors can contribute to this plasticity of phenotypes, namely the antibody-dependent enhancement (ADE) [6]. A patient infected for the first time with a DENV serotype becomes immunized against that serotype and usually develops DF in a milder form; if a secondary infection by another serotype occurs, the previously formed memory T-cells are activated, being ineffective in combatting the new serotype, and leading to the production of high concentrations of inflammatory cytokines (IFN-γ, TFN-α and IL-13) and a low concentration of anti-inflammatory ones (IL-10). Consequently, the patient usually develops more aggressive phenotypes (DHF or even DSS). Host genetic factors are also associated with susceptibility to DENV, including immune system factors such as HLA-I and HLA-II, TNF-α, Fc receptor, TAP and DC-SIGN (reviewed in [7]); immune system and endothelial homeostasis genes *MICB* and *PLCE1* [8]; genes controlling lipid (*OSBPL10* and *RXRA* [9]) and xenobiotic metabolism (*PLCB4*, *CHST10*, *AHRR*, *PPP2R5E* and *GRIP1* [10]). A metanalysis of several of these genes across worldwide populations [11] made it possible to infer a preliminary worldwide map of host susceptibility to Dengue disease: sub-Saharan Africans and descendants are best protected against severe forms; Europeans and close neighbours are best protected against DF but not against severe forms; Northeast and Southeast Asians are less protected against mild and severe forms.

Dengue disease symptoms reflect the various alterations occurring after infection in key organs like the liver, spleen and encephalon, and further studies are needed to fully elucidate the local mechanisms. The liver displays signs of auto-immune response, circulatory compromising, hypoxia and hypotension caused by vascular leakage, hepatomegaly and a rise of transaminase enzyme levels [12,13]. Sudden spleen ruptures have been reported in the early acute and convalescent phases, which can be fatal if not treated immediately through surgery [14]. A comparison of transcriptomic profiles between infected and control groups provides meaningful insights into the human and pathogen interactions in diverse tissues and may help in understanding the pathophysiology of the disease. Regarding Dengue, a few transcriptomic analyses have been conducted in Asian and South American populations for diverse phenotypes or phases of the disease, but all were in blood samples or in cancer cell lines and not in tissues from patients [15,16,17,18,19,20]. It would be very informative to analyze Dengue-affected tissues, since the transcriptome profiles differ with tissue/organ specialization.

No antiviral treatment is available against Dengue infection, and, essentially, infected patients receive treatment to minimize and manage the symptoms [21]: antipyretics are used to reduce pain and fever in mild cases; crystalloids are used to maintain intravascular volume, blood pressure and normal urine formation; and volume expanders are used for the restauration of intravascular volume, blood pressure and tissue perfusion in DHF and DSS. Treatment with immunomodulators, corticosteroids and other nonsteroidal anti-inflammatory drugs has shown no results and should be avoided [21]. Statins, a group of drugs known as HMG-CoA reductase inhibitors, which are administrated to patients with hyperlipidaemia, have been investigated in the Dengue context, given the lipid metabolism importance in viral infections and their effect on the endothelial function. Promising in vitro (reduced DENV virion assembly; [22]) and in vivo (increased survival rate and decreased DENV viremia in DENV-infected mice treated with Lovastatin; [23]) results were unfortunately not replicated in clinical trials [24,25]: statins were well tolerated by infected patients but provided no beneficial effects in reducing symptoms or viremia. Disappointingly, the Sanofi Pasteur vaccine, which had been introduced in some endemic countries, had its general use hampered by fatalities of children with severe Dengue in the Philippines [26]. 

Recent developments in mining big data are revolutionizing the development and repurposing of drugs [27], making it possible to narrow down candidate drugs to be tested, and leading to significant reductions in time and costs. Drug repurposing, the process of finding new uses for approved pharmaceutical drugs or investigational compounds by relying on the biological targets shared between diseases and the pleiotropic actions of drugs, further simplifies the process. In fact, as these repurposed drugs have already been tested in humans, doses and side effects are known. The current COVID-19 pandemic testifies to the high impact of this type of approach [28]. CMap is an example of a mining tool for in silico drug discovery that was built on genetic expression profiles from tissues treated with many molecules [29]. The second phase of CMap, called L1000 [30], includes 1,319,138 profiles from 42,080 perturbagens, corresponding to 25,200 biological entities for a total of 473,647 signatures. Users can input information on up- and downregulated genes in a certain disease into the CMap tool and obtain the drugs related to that gene expression pattern via data mining and pattern searching algorithms. Successful examples of CMap use have been published in cancer research [31,32]. In Dengue [33], CMap analysis of blood transcriptomics was combined with other miners on blood-based proteomic and protein–protein interaction datasets, making it possible to infer eight candidate drugs: estradiol, etoposide, simvastatin, resveratrol, sirolimus, valproic acid, vorinostat and Y-27632 compound (kinase inhibitor).

In this work, we aimed to identify drugs that could be effective against Dengue disease by applying a multi-tissue transcriptomic-informed in silico investigation. First, we analyzed the transcriptome from liver (published data), spleen (our own data) and blood (published). We checked for significantly differentially expressed genes in the pairwise comparisons and evaluated which molecular pathways were significantly changed. Second, we evaluated through CMap which drugs could interfere with the expression profiles from each of the tissues in the Dengue context.

## 2. Materials and Methods

### 2.1. Biological Samples, Laboratorial Processing and Raw Data Analysis

Tissue samples from liver, spleen and encephalon were post-mortem collected from deceased Cuban individuals that died from Dengue (case cohort: IDs 875, 900, 37478, 39538 and 39539; cases 875 and 900 did not include encephalon sample) or traffic accidents (control cohort obtained at the Cuban Institute of Legal Medicine during routine autopsy examination: IDs F1, F2 and F4). Additional information on the individuals is included in Table S1. The study was approved 20 February 2018 by the Institutional Ethical Review Committee of the Institute of Tropical Medicine Pedro Kourí (IPK), with the number CEI-IPK 30-18, for studies with Arbovirus with medical importance in Cuba, 2017–2021, including Dengue fatal cases and controls. The consent for using tissue samples in this research was verbally provided by relatives of the deceased cases prior to the necropsy. This consent procedure was also included in the ethics approval. Tissue fragments sized 5 × 5 mm^3^ (20–30 mg approximately) were collected and immersed in RNAlater at 4 °C overnight, then stored at −80 °C the following day. The frozen tissues from Dengue-deceased cases arrived at the IPK’s National Reference Laboratory for Arbovirus 72 h after the patient’s death, while the control cases arrived 24 h after death. For RNA isolation, tissues were disrupted using the Tissue Lyser II kit (QIAGEN), and RNA was extracted using a column-based method (RNeasyProtect Mini kit, Qiagen, Hilden, Germany). For the DENV detection in tissue samples, two methods were used: a conventional capsid and premembrane gene (C-prM) RT-PCR protocol for samples from 2014 [34] and the CDC Dengue multiplex real-time qRT-PCR assay (four serotypes) targeting the capsid (C) gene [35].

Gene expression evaluation was done by Next Generation Sequencing (NGS), using the Ion AmpliSeq Transcriptome Human Gene Expression Kit, Ion 550TM Chip kit and Ion S5TM XL System (Thermo Fisher Scientific, Waltham, MA, USA). This kit contains one amplicon per 20,812 protein-coding genes. The total raw reads allowed in the chip for each sample were around 10.8 million reads.

Raw fastq files were checked for quality control using FastQC software, by evaluating the overall sequence quality scores, the base sequence content, the sequence GC content and the presence of duplicated or overrepresented sequences. Low-quality bases were further filtered by a sliding window (four nucleotides with a mean average below 15). A minimum length of 40 bases was set to decrease multi-mapping. Alignment was performed in Bowtie2 with the “local-sensitive” flag against the human hg19 reference. After alignment, reads were sorted and converted using samtools [36] and counted using HTSeq, exclusively for the amplified regions.

### 2.2. Published Datasets

Two microarray-based transcriptomic datasets from Dengue-infected patients and healthy controls were retrieved from the Gene Expression Omnibus (GEO) database [37], identified by IDs GSE18090 [15] and GSE51808 [19]. GSE18090 contained data from Peripheral Blood Mononuclear Cells (PBMCs) collected from Brazilian adults, while GSE51808 referred to whole blood samples from Thai children and adults. The arrays used for GSE18090 and GSE51808 were Affymetrix Human Genome U133 Plus 2.0 and Affymetrix HT HG-U133+ PM Array Plate, respectively. Samples included in the analysis belonged to DHF patient and healthy control groups; convalescent individuals (only in the GSE51808 dataset) and DF (milder form) were excluded from analysis. Outlier samples in both datasets were removed after principal component analysis (PCA; three samples for GSE18090 and two samples for GSE51808). Final numbers of individuals were seven controls and eight DHF for GSE18090 and nine controls and eight DHF for GSE51808. As the microarrays have multiple probes per gene, data were filtered in R with the “genefilter” package featureFilter function (which keeps the probe with the highest expression variance for each gene).

A publicly available viscRNA-Seq dataset (virus-including single cell RNA-seq) [38], referring to the transcriptome of individual human hepatoma (Huh7) cells infected by DENV, was downloaded from GEO (ID: GSE110496). The two groups considered for analyses were non-infected (after quality control, 67 single cells were considered) and infected at an MOI (multiplicity of infection) of 10 and collected after 48 h of infection (75 single cells included).

### 2.3. Differential Expression Evaluation and Gene Set Enrichment Analysis

For the AmpliSeq dataset, differential expression analysis between the infected and controls was carried for each tissue individually, using the DESeq2 R package [39]. DESeq2 applies a negative binomial distribution to model gene counts and test for differential expression.

Gene expression profiles from the microarray datasets were normalized through the Robust Multi-array Average (RMA) algorithm using the R oligo package. To increase sensitivity, low-intensity probes were removed from the normalized probe intensity values. Probes were then collapsed to the genes for gene differential expression (DE) analysis, using functions from the limma package [40].

Raw single cell counts were analyzed by the SingleCellExperiment R package. The data were filtered using the following parameters: gene present in >3 cells, cell with >4000 genes, total number of counts above 1 × 10^5^, and percent of mitochondrial genes <10%. After filtering, counts were log normalized, and a tSNE was performed with a perplexity of 10 for the identification and removal of visual outliers. Raw counts from single cells that passed these filters were loaded into DESeq2 [39] and processed as for the Ampliseq dataset.

An adjusted *p*-value < 0.05 was used for attributing a differentially expressed gene status. Enrichment analysis was done using GSEA [41] for Gene Ontology (CC, BP and MF) and KEGG pathway databases. The pre-ranking option was used, taking into account an ordered list of genes for the differential expression. Volcano plots for the expression profiles were created using the R packages “ggplot2” and “ggrepel”. Venn diagrams with common upregulated and downregulated genes between datasets were generated in “VennDiagram” R package.

### 2.4. Drug Repurposing

For the drug repurposing step, the CMap Query tool from clue.io (https://clue.io/query; accessed on 27 January 2021) was used. For the blood information, the first 150 (this number is a limitation from the tool) genes that were significantly up- and down-regulated (false discovery rate, FDR values) between patients and controls, in at least one dataset, were uploaded in an ordered way according to the highest absolute mean fold change value across the two datasets. In the case of the AmpliSeq and Huh7 single cell data, query analyses were made independently for each tissue, inputting the 150 upregulated and downregulated genes ranked by the lowest *p*-values (even if they were not statistically significant).

We queried the CMap Touchstone dataset containing perturbation data for 2837 compounds tested in nine human cell lines: A375 (malignant melanoma), A549 (non-small cell lung carcinoma), HCC515 (non-small cell lung adenocarcinoma), HEPG2 (hepatocellular carcinoma cell line), MCF7 (breast adenocarcinoma), PC3 (prostate adenocarcinoma), VCAP (metastatic prostate cancer), HT29 (colorectal adenocarcinoma), and HA1E (kidney epithelial immortalized). The CMap query output consists of a list of perturbagens rank-ordered by the similarity of differentially expressed gene sets to the query gene set. These results come in the form of a connectivity map score, tau (τ), ranging from −100 to 100, which compares the observed enrichment score seen in the inputted data with all others in the reference database. τ applies a weighted connectivity score based on the weighted Kolmogorov–Smirnov enrichment statistic, normalized across cell types and perturbations. A τ of 90 indicates that only 10% of reference perturbations showed stronger connectivity to that query. A positive score indicates there is similarity between a given perturbagen’s signature and that of the query, while a negative score indicates that the two signatures are opposing. Thus, we considered scores of below −90 as drugs that could potentially treat Dengue disease. The drugs are complemented with information for their molecular mechanism of action. We then classified these drugs into seven main groups of actions: antineoplastic, antibiotic, antiparasitic, immunosuppressant, cardiovascular, anti-inflammatory, antiviral and other. This classification was based on information contained in several databases: Inxight: Drugs (https://drugs.ncats.io/; accessed on 27 January 2021); PubChem [42]; Drugbank [43]; FDA (https://www.fda.gov/; accessed on 27 January 2021); EMA (https://www.ema.europa.eu/en; accessed on 27 January 2021); ClinicalTrials (https://clinicaltrials.gov/; accessed on 27 January 2021). Some molecular mechanisms of action can be affiliated to more than one main group of action. The bar plots created for demonstrating the distribution of drugs among different groups of action were created using the “ggplot2” package from R.

## 3. Results

### 3.1. Expression Profiles in the Liver, Spleen and Encephalon Dengue Cohorts

The AmpliSeq characterization of the extracted RNA from the tissues aimed at approximately 10 million reads per sample, but the initial amount of reads obtained was lower, varying between 1,410,487 and 9,239,028, with a mean of 5,412,203. There was no effect of the tissue analyzed, as the mean of initial reads was similar between tissues: 5,987,967 in spleen, 5,136,132 in encephalon and 5,077,912 in liver. An initial quality control, based on the distribution of GC content, showed that the available tissue samples (liver and spleen) from cases 900 and 875 did not follow the theoretical unimodal distribution, presenting bimodal distributions. Since bimodal distributions are a common indicator of some form of contamination, these samples were removed from further analyses.

To evaluate if the clustering of samples mirrored the tissue of origin, a PCA was conducted (Supplementary Figure S1). Spleen samples were clustered, with some differentiation between infected and control samples, but encephalon and liver samples presented a high variance, with no clustering with infection status. The time elapsing between death and collection of tissue may have interfered with the RNA quality in encephalon and liver, which are known to degrade fast [44]. We conducted a bibliographic search in order to check whether there were transcriptome datasets that had been published that could be compared with/replace our tissue data for encephalon and liver, and verified that a recent study was available only for a liver cell line infected by DENV [38]. We decided to proceed with the analysis of our tissue data for spleen, replace the tissue data for liver with the data available from the in vitro infection of the liver cell line, and remove encephalon from the analysis.

The differential expression analyses revealed the following values of significantly upregulated and downregulated genes (Supplementary Figure S2; Supplementary Tables S2 and S3): 23 and 89 in spleen and 1069 and 1319 in the liver single cell dataset. A careful survey of the top differentially expressed genes and pathways in the two tissues indicated signals that make sense in the context of infection, supporting the reliability of the results obtained here.

**Figure 1:**
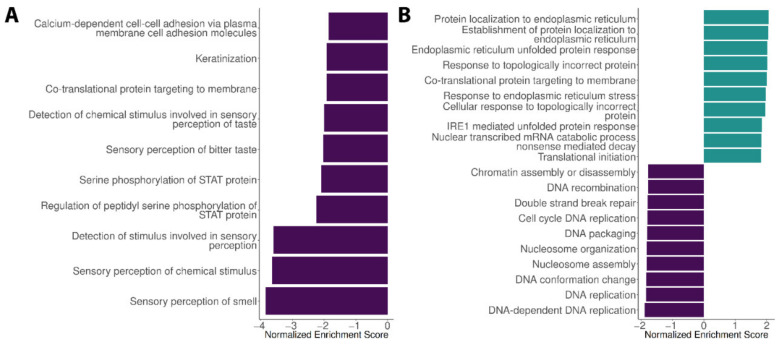
Top 20 Gene Ontology Biological Process (GO-BP) pathways in the (**A**) spleen and (**B**) liver cells (cut-off values: nominal *p*-value < 0.05 and FDR < 0.25). Positive normalized enrichment score (NES; in purple) represents upregulated pathways in the infected individuals versus controls, while negative NES values (in green) represent downregulated pathways in the infected individuals versus controls.

The spleen tissue of the infected patients showed upregulation of the *AVPR2* gene (vasopressin receptor), which increases retention of water by increasing cyclic AMP [45], and the *TYRO3* gene, which activates the AKT survival pathway and mediates the clearance of apoptotic cells [46]. Pathway enrichment analysis for the spleen tissue (Figure 1A; Supplementary Figures S3–S5) revealed a downregulation of immune components related with the phosphorylation of the STAT protein, type I interferon, autophagy and RIG-I-like receptor signaling pathway; the latter is a target of DENV that regulates RIG-I-directed IFN induction to successfully replicate and spread [47]. Dimerization of STATs is essential for the establishment of classical JAK-STAT signaling pathways, which have an important role in the control of immune responses. A downregulation of signaling pathways related to the response to angiotensin was also detected. On the other hand, the upregulation of insulin and cholesterol metabolic pathways could also be distinguished.

In the liver cells, we noticed an upregulation of *ATF3*, a activating transcription factor 3, which is a suppressor of pro-inflammatory responses [48] and has been considered a hub of the cellular adaptative response [49]. Other transcription factors responsible for the pro-inflammatory response, such as *TNFRSF9* and *MXD1*, were also found upregulated. Pathway enrichment analysis in the liver cells (Figure 1B; Supplementary Figures S3–S5) showed a downregulation of cell cycle pathways, an observation previously made [38]. Hepatic manifestations may be the result of cell cycle arrest, which through the inhibition of cell death may allow immune evasion and/or help promote viral assembly. In the literature, it has been shown that HepG2 (liver) cells were significantly more permissive for both infection and virus production in the G(2) phase than Vero (kidney) cells [50].

### 3.2. Expression Profiles in the Blood Dengue Cohorts

**Figure 2:**
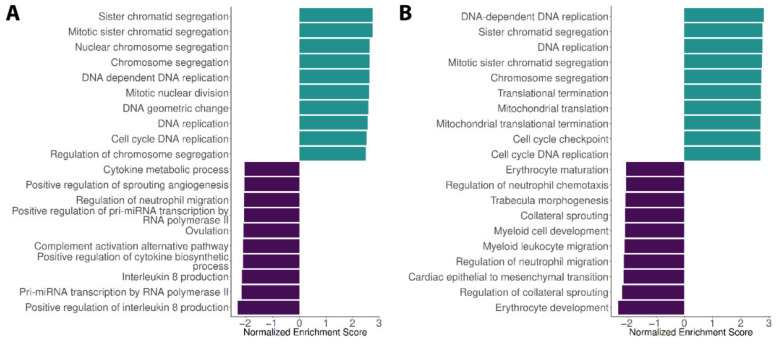
Top 20 Gene Ontology Biological Process (GO-BP) in the two blood datasets: (**A**) GSE18090 and (**B**) GSE51808 pathways (cut-off values: nominal *p*-value < 0.05 and FDR < 0.25). Positive NES (in purple) represents upregulated pathways in the infected individuals versus controls, while negative NES values (in green) represent downregulated pathways in the infected individuals versus controls.

The blood Dengue cohorts showed a strong partition in gene expression between the infected and control clusters (Supplementary Figure S6). Several genes were differentially expressed (Supplementary Figure S7; full information provided in Supplementary Tables S4 and S5), especially in the GSE51808 dataset from whole blood (9230 differentially expressed genes), in contrast with GSE18090 from PBMCs only (311). The heterogeneity in the original blood cells could explain the discrepancy between datasets. When applying Venn diagrams to the upregulated and the downregulated sets of these genes (Supplementary Figure S8), 208 upregulated and 54 downregulated genes were shared between datasets. To investigate which type of pathways were upregulated and downregulated, gene set enrichment analysis was conducted in each dataset. Figure 2 represents the top-20 significantly enriched pathways, when using the database KEGG (results with GO database significantly enriched pathways are reported in Supplementary Figures S9–S11). As can be observed in the figure, the infected group displayed downregulation of biological pathways related with the immune system and response to pathogens, and upregulation in biological pathways related with cell cycle and repair mechanisms, an observation previously made for PBMC samples in Nicaraguan children [17].

### 3.3. Drug Repurposing

The CMap tool results for the drug discovery in the blood cohort indicated a total of 203 known compounds that were inferred (CMap score inferior to −90) as having a potential impact in Dengue hemorrhagic fever treatment (Supplementary Table S6). These drugs were spread across seven main different mechanisms of action: antineoplastic (120), antibiotic (11), antiparasitic (2), immunosuppressant (6), cardiovascular (11), anti-inflammatory (9) and antiviral drugs (7). Among these, some compounds have already been shown to have some action against DENV or the mechanism of action taken by flavivirus. Some examples are the purvalanol A and palbociclib, both cyclin-dependent kinase inhibitors, which have shown anti-flavivirus activity [51]. Mycophenolic acid, a drug currently used as an immunosuppressive agent, has been shown to inhibit flavivirus infection by preventing synthesis and accumulation of viral RNA [52].

**Figure 3:**
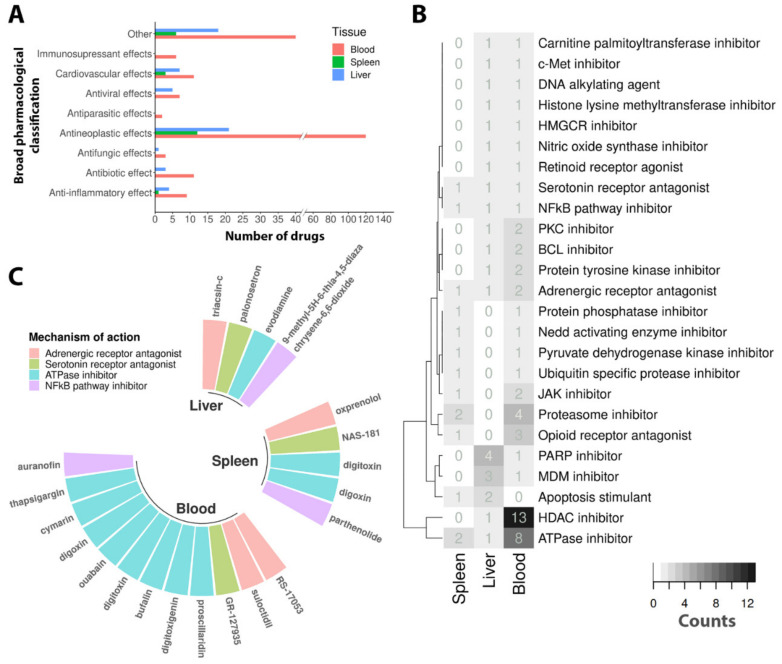
CMap-identified compounds (score equal to or below −90) that potentially impact Dengue haemorrhagic fever treatment, according to their mechanism of action in the blood, spleen and liver. (**A**) Bar plot of the distribution of drugs by broad pharmacological classification; (**B**) heatmap of drugs per mechanism identified in one or more tissues; (**C**) circus plot of common mechanisms of action.

When analyzing the CMap results for all tissues included in this work (Figure 3; Supplementary Tables S7 and S8), we observed a similar pattern as the one identified in the blood, with some variations between tissues. Forskolin, an antihypertensive agent and a platelet aggregation inhibitor with an anti-viral effect against HIV [53], also detected in the liver, was the only drug observed in more than one tissue. Norethindrone is used as a contraceptive, and it might confer some protection against Dengue, since pregnant women have an increased risk of developing severe Dengue infections [54]. In the liver, atorvastatin (statin), dexamethasone (corticosteroids) and prostaglandin A1 (anti-viral) has shown effects on cholesterol metabolism, immune response, and viral replication, respectively. In the spleen, geldanamycin, an inhibitor of Hsp90, which has a role in viral replication, has shown some effect in the reduction of Dengue viral infection [55].

Given the high stochasticity in identifying a drug from a connectivity map including 1,000,000 profiles, we instead based the comparison between tissues on the CMap molecular mechanism of action. Four mechanisms of action were found to be common to all tissues: “Adrenergic receptor antagonist”, “ATPase inhibitor”, “NF-kB pathway inhibitor” and “Serotonin receptor antagonist”.

## 4. Discussion

The gene expression profiles that have been perturbed pharmacologically and have been obtained in vitro, such as the second phase of CMap, known as L1000 [30], are beginning to shed light on gene function and on understanding the molecular basis of diseases [56]. A first attempt was conducted on blood expression profiles of Dengue patients against CMap [33], but a broader picture for other affected tissues was needed and seemed a good aim to pursue in this work. A first step consisted of obtaining post mortem human tissue samples for studying the patterns of gene expression underlying tissue specificity, as sampling many tissues from living individuals would be impossible. However, a drawback of the post mortem human tissue samples is the significant reduction of RNA, which degrades quickly upon death [57]. As we saw in this work, despite the careful steps we took in collecting the samples as soon as possible after autopsies, degradation affected the liver and encephalon tissues, leading us to exclude the transcriptomic data for these tissues.

By referring to our own transcriptomic dataset on the spleen and complementing with published datasets for the blood and liver of Dengue patients, we identified some heterogeneity in molecular pathways between tissues. The dissimilarity in the results reflecting tissue specificity could initially indicate that a cocktail-based treatment would be more suitable to address this diversity. However, given the high connectivity between molecular networks in the body, and the multi-effects of drugs, some candidate drugs can be effective in the various tissues for Dengue disease. Notably, drugs sharing the mechanism of actions in the “NF-kB pathway inhibitor”, “ATPase inhibitor”, “Adrenergic receptor antagonist” and “Serotonin receptor antagonist” seem to be promising candidates in Dengue treatment, and some previous pin-point functional tests have contributed evidence supporting the antiviral action of some of these mechanisms. By pharmacologically inhibiting NF-kB activation, Cheng et al. [58] observed that the potential role of NF-kB in oxidative signaling is prevented during Dengue infection through the abolishment of iNOS/NO biosynthesis and TNF-α production. Interestingly, the NF-kB pathway inhibitor compounds identified in this work, auranofin and parthenolide, have previously been shown to successfully inhibit flavivirus infection [59,60]. ATPase inhibitors, such as evodiamine, digoxin and digitoxin, also present strong anti-inflammatory responses. Evodiamine inhibits nitric oxide production by interfering with the interferon-gamma and inhibiting the action of NF-kB signal events [61]. The impact of evodiamine in the inhibition of viral replication, much like digitoxin, is still limited to influenza A [62,63]. Digitoxin administration to influenza A virus (IAV)–infected cotton rats has been shown to lower pro-inflammatory cytokine levels of TNFα, GRO/KC, MIP2, MCP1, and IFN-γ in lungs, commonly associated with cytokine storm [63]. Since high levels of inflammatory cytokines are observed in severe Dengue infection, digitoxin might have a therapeutic potential. Besides digitoxin, digoxin, another cardiac glycoside, displays antiviral effects against Zika virus [59]. Other authors [64] have identified a group of small molecules that inhibit infection with Dengue and other flaviviruses, and interestingly those molecules were similar in structure to tricyclic antipsychotic compounds, which act as antagonists of serotonin and dopamine receptors. Further knockdown of dopamine receptor D4 reduced DENV replication via inhibition of epidermal growth factor receptor (EGFR)–related kinase (ERK) phosphorylation. Although the involvement of receptors that are mainly located in neurons seems unexpected, it has been shown that dopamine receptors are expressed on rodent and human macrophages, a primary target cell of DENV infection. These macrophage-related dopamine receptors may be the ones being influenced by DENV.

Other mechanisms seem promising druggable targets in certain tissues. This is the case for the inhibition of histone deacetylases (HDAC) in blood, as this class of enzymes is essential in normal hematopoiesis, namely in the cell differentiation and proliferation [65]. The HDAC inhibitor valproic acid has been previously shown to downregulate cytokine expression on Dengue-infected macrophages [66]; the CMap tool identified this drug both in our results and in another data mining work [33]. The signal we detected in spleen and blood for the proteasome inhibition was previously demonstrated functionally [67] in human monocytic cells and in mice infected with DENV; the drug acting in this mechanism inhibited infectious DENV production in primary monocytes and the spread of DENV in the spleen. Poly (ADR-ribose) polymerase (PARP) seems to be a key mediator of liver inflammation and fibrosis [68], suggesting the possible process by which a PARP inhibitor would be effective in controlling DENV in the liver, as identified here.

At first instance, there were not many indications of lipid-related drugs in the CMap results. This may be because of the late stage of the disease analyzed in this work, while lipids may be more important in the first stages of entrance of the virus into the cells and in their replication in the acute phase [9]. Even so, a “LXR agonist” mechanism was identified in the spleen, and statins (atorvastatin and simvastatin, respectively) were highlighted in the liver and blood. These results call for a continuation of trials with statins (bigger sample sizes; different concentrations of intake; different times of intake), as the few clinical trials already performed [24,25] showed that treatment or continuation of statin intake has no negative effects on Dengue patients.

The development of a new drug takes several years before it can become accessible for treatment of patients or prophylactic protection. On the other hand, repurposed drugs can skip several steps in the process of approval and commercialization, as safety, toxicity and risk assessment tests have already been conducted in human clinical trials. This second class of drugs can be highly valuable for a faster battle against Dengue disease and its complications, decreasing the pressure due to the increasing incidence of DENV infection and its rapid spread across the world.

## 5. Conclusions

Our results showed that despite some tissue heterogeneity in the response to DENV, drugs acting on “Adrenergic receptor antagonist”, “ATPase inhibitor”, “NF-kB pathway inhibitor” and “Serotonin receptor antagonist” mechanisms would be effective across tissues. Some of these candidate drugs have been pinpointedly described in the literature to have an impact against DENV or other viruses affiliated with the flavivirus family. Our work advances considerably the knowledge in this area by demonstrating that these four molecular mechanisms can be pharmaceutically modulated across the tissues most affected by Dengue disease.

## Data Availability

Raw data are publicly available at the European Nucleotide Archive (ENA), with Accession Number PRJEB46564.

## Supplementary Materials

The following are available online at https://www.mdpi.com/article/10.3390/v13081540/s1, Table S1: Additional information on the individuals from whom samples were collected for transcriptomics; Figure S1: PCA plot (PC1 explaining 81.4% of variation vs. PC2 explaining 6.2% of variation) of the human transcriptomic profile of the spleen, liver and encephalon tissues; Figure S2: Volcano plots for the gene differential expression in the (A) spleen and (B) liver cell datasets; Table S2: Differentially expressed genes between infected and control cohorts for the spleen tissue samples (adjusted *p*-value < 0.05); Table S3: Differentially expressed genes between infected and control cohorts for the liver single cell dataset (adjusted *p*-value < 0.05); Figure S3: Top 20 Gene Ontology Cellular Component (GO-CC) pathways in the (A) spleen and (B) liver cells; Figure S4: Top 20 Gene Ontology Molecular Function (GO-MF) pathways in the (A) spleen and (B) liver cells; Figure S5: Top 20 significantly enriched KEGG pathways in the (A) spleen and (B) liver cells; Figure S6: Dendrograms for the gene expression profiles in the two blood datasets: (A) GSE18090 and (B) GSE51808; Figure S7: Volcano plots for the gene differential expression in the two blood datasets: (A) GSE18090 and (B) GSE51808; Table S4: Differentially expressed genes between infected and control cohorts for the GSE18090 dataset (adjusted *p*-value < 0.05); Table S5: Differentially expressed genes between infected and control cohorts for the GSE51808 dataset (adjusted *p*-value < 0.05); Figure S8: Venn diagrams of the upregulated (A) and downregulated (B) genes in the two blood datasets; Figure S9: Top 20 Gene Ontology Cellular Component (GO-CC) pathways in the (A) GSE18090 and (B) GSE51808; Figure S10: Top 20 Gene Ontology Molecular Function (GO-MF) pathways in the (A) GSE18090 and (B) GSE51808; Figure S11: Top 20 significantly enriched KEGG pathways in the (A) GSE18090 and (B) GSE51808; Table S6: CMap results for the blood microarray data; Table S7: CMap results for the spleen tissue data; Table S8: CMap results for the liver cell data.

## References

[1] Guzman M.G., Gubler D.J., Izquierdo A., Martinez E., Halstead S.B. (2016). Dengue infection. Nat. Rev. Dis. Primers.

[2] Mayer S.V., Tesh R.B., Vasilakis N. (2017). The emergence of arthropod-borne viral diseases: A global prospective on dengue, chikungunya and zika fevers. Acta Trop..

[3] Bhatt S., Gething P.W., Brady O.J., Messina J.P., Farlow A.W., Moyes C.L., Drake J.M., Brownstein J.S., Hoen A.G., Sankoh O. (2013). The global distribution and burden of dengue. Nature.

[4] Shepard D.S., Undurraga E.A., Halasa Y.A., Stanaway J.D. (2016). The global economic burden of dengue: A systematic analysis. Lancet Infect. Dis..

[5] Kraemer M.U., Sinka M.E., Duda K.A., Mylne A.Q., Shearer F.M., Barker C.M., Moore C.G., Carvalho R.G., Coelho G.E., Van Bortel W. (2015). The global distribution of the arbovirus vectors Aedes aegypti and Ae. albopictus. Elife.

[6] Halstead S.B., Mahalingam S., Marovich M.A., Ubol S., Mosser D.M. (2010). Intrinsic antibody-dependent enhancement of microbial infection in macrophages: Disease regulation by immune complexes. Lancet Infect. Dis..

[7] Oliveira M., Ferreira J., Fernandes V., Sakuntabhai A., Pereira L. (2018). Host ancestry and dengue fever: From mapping of candidate genes to prediction of worldwide genetic risk. Future Virol..

[8] Khor C.C., Chau T.N., Pang J., Davila S., Long H.T., Ong R.T., Dunstan S.J., Wills B., Farrar J., Van Tram T. (2011). Genome-wide association study identifies susceptibility loci for dengue shock syndrome at MICB and PLCE1. Nat. Genet..

[9] Sierra B., Triska P., Soares P., Garcia G., Perez A.B., Aguirre E., Oliveira M. (2017). OSBPL10, RXRA and lipid metabolism confer African-ancestry protection against dengue haemorrhagic fever in admixed Cubans. PLoS Pathog..

[10] Oliveira M., Lert-Itthiporn W., Cavadas B., Fernandes V., Chuansumrit A., Anunciação O., Casademont I., Koeth F., Penova M., Tangnararatchakit K. (2018). Joint ancestry and association test indicate two distinct pathogenic pathways involved in classical dengue fever and dengue shock syndrome. PLoS Negl. Trop. Dis..

[11] Oliveira M., Saraiva D.P., Cavadas B., Fernandes V., Pedro N., Casademont I., Koeth F., Alshamali F., Harich N., Cherni L. (2018). Population genetics-informed meta-analysis in seven genes associated with risk to dengue fever disease. Infect. Genet. Evol..

[12] Trung D.T., Thao L.T.T., Hien T.T., Hung N.T., Vinh N.N., Hien P.T., Chinh N.T., Simmons C., Wills B. (2010). Liver involvement associated with dengue infection in adults in Vietnam. Am. J. Trop. Med. Hyg..

[13] Samanta J., Sharma V. (2015). Dengue and its effects on liver. World J. Clin. Cases.

[14] de Silva W.T., Gunasekera M. (2015). Spontaneous splenic rupture during the recovery phase of dengue fever. BMC Res. Notes.

[15] Nascimento E.J., Braga-Neto U., Calzavara-Silva C.E., Gomes A.L., Abath F.G., Brito C.A., Cordeiro M.T., Silva A.M., Magalhães C., Andrade R. (2009). Gene expression profiling during early acute febrile stage of dengue infection can predict the disease outcome. PLoS ONE.

[16] Loke P., Hammond S.N., Leung J.M., Kim C.C., Batra S., Rocha C., Balmaseda A., Harris E. (2010). Gene expression patterns of dengue virus-infected children from nicaragua reveal a distinct signature of increased metabolism. PLoS Negl. Trop. Dis..

[17] Popper S.J., Gordon A., Liu M., Balmaseda A., Harris E., Relman D.A. (2012). Temporal dynamics of the transcriptional response to dengue virus infection in Nicaraguan children. PLoS Negl. Trop. Dis..

[18] Sessions O.M., Tan Y., Goh K.C., Liu Y., Tan P., Rozen S., Ooi E.E. (2013). Host cell transcriptome profile during wild-type and attenuated dengue virus infection. PLoS Negl. Trop. Dis..

[19] Kwissa M., Nakaya H.I., Onlamoon N., Wrammert J., Villinger F., Perng G.C., Yoksan S., Pattanapanyasat K., Chokephaibulkit K., Ahmed R. (2014). Dengue virus infection induces expansion of a CD14(+)CD16(+) monocyte population that stimulates plasmablast differentiation. Cell Host Microbe.

[20] Long H.T., Hibberd M.L., Hien T.T., Dung N.M., Van Ngoc T., Farrar J., Wills B., Simmons C.P. (2009). Patterns of gene transcript abundance in the blood of children with severe or uncomplicated dengue highlight differences in disease evolution and host response to dengue virus infection. J. Infect. Dis..

[21] Rajapakse S., Rodrigo C., Rajapakse A. (2012). Treatment of dengue fever. Infect Drug Resist..

[22] Martínez-Gutierrez M., Castellanos J.E., Gallego-Gómez J.C. (2011). Statins reduce dengue virus production via decreased virion assembly. Intervirology.

[23] Martinez-Gutierrez M., Correa-Londoño L.A., Castellanos J.E., Gallego-Gómez J.C., Osorio J.E. (2014). Lovastatin delays infection and increases survival rates in AG129 mice infected with dengue virus serotype 2. PLoS ONE.

[24] Whitehorn J., Nguyen C.V.V., Khanh L.P., Kien D.T.H., Quyen N.T.H., Tran N.T.T., Hang N.T., Truong N.T., Hue Tai L.T., Cam Huong N.T. (2016). Lovastatin for the Treatment of Adult Patients With Dengue: A Randomized, Double-Blind, Placebo-Controlled Trial. Clin. Infect. Dis..

[25] Chia P.Y., Htun H.L., Ling W.P., Leo Y.S., Yeo T.W., Lye D.C.B. (2018). Hyperlipidemia, statin use and dengue severity. Sci. Rep..

[26] Wilder-Smith A., Flasche S., Smith P.G. (2019). Vaccine-attributable severe dengue in the Philippines. Lancet.

[27] Loging W., Harland L., Williams-Jones B. (2007). High-throughput electronic biology: Mining information for drug discovery. Nat. Rev. Drug. Discov..

[28] Gordon D.E., Hiatt J., Bouhaddou M., Rezelj V.V., Ulferts S., Braberg H., Jureka A.S., Obernier K., Guo J.Z., Batra J. (2020). Comparative host-coronavirus protein interaction networks reveal pan-viral disease mechanisms. Science.

[29] Lamb J., Crawford E.D., Peck D., Modell J.W., Blat I.C., Wrobel M.J., Lerner J., Brunet J.P., Subramanian A., Ross K.N. (2006). The Connectivity Map: Using gene-expression signatures to connect small molecules, genes, and disease. Science.

[30] Subramanian A., Narayan R., Corsello S.M., Peck D.D., Natoli T.E., Lu X., Gould J., Davis J.F., Tubelli A.A., Asiedu J.K. (2017). A Next Generation Connectivity Map: L1000 Platform and the First 1,000,000 Profiles. Cell.

[31] Agren R., Mardinoglu A., Asplund A., Kampf C., Uhlen M., Nielsen J. (2014). Identification of anticancer drugs for hepatocellular carcinoma through personalized genome-scale metabolic modeling. Mol. Syst. Biol..

[32] Uhlen M., Zhang C., Lee S., Sjöstedt E., Fagerberg L., Bidkhori G., Benfeitas R., Arif M., Liu Z., Edfors F. (2017). A pathology atlas of the human cancer transcriptome. Science.

[33] Amemiya T., Gromiha M.M., Horimoto K., Fukui K. (2019). Drug repositioning for dengue haemorrhagic fever by integrating multiple omics analyses. Sci. Rep..

[34] Lanciotti R.S., Calisher C.H., Gubler D.J., Chang G.J., Vorndam A.V. (1992). Rapid detection and typing of dengue viruses from clinical samples by using reverse transcriptase-polymerase chain reaction. J. Clin. Microbiol..

[35] Santiago G.A., Vergne E., Quiles Y., Cosme J., Vazquez J., Medina J.F., Medina F., Colón C., Margolis H., Muñoz-Jordán J.L. (2013). Analytical and clinical performance of the CDC real time RT-PCR assay for detection and typing of dengue virus. PLoS Negl. Trop. Dis..

[36] Li H., Handsaker B., Wysoker A., Fennell T., Ruan J., Homer N., Marth G., Abecasis G., Durbin R. (2009). The Sequence Alignment/Map format and SAMtools. Bioinformatics.

[37] Barrett T., Wilhite S.E., Ledoux P., Evangelista C., Kim I.F., Tomashevsky M., Marshall K.A., Phillippy K.H., Sherman P.M., Holko M. (2013). NCBI GEO: Archive for functional genomics data sets--update. Nucleic Acids Res..

[38] Zanini F., Pu S.Y., Bekerman E., Einav S., Quake S.R. (2018). Single-cell transcriptional dynamics of flavivirus infection. Elife.

[39] Love M.I., Huber W., Anders S. (2014). Moderated estimation of fold change and dispersion for RNA-seq data with DESeq2. Genome Biol..

[40] Ritchie M.E., Phipson B., Wu D., Hu Y., Law C.W., Shi W., Smyth G.K. (2015). limma powers differential expression analyses for RNA-sequencing and microarray studies. Nucleic Acids Res..

[41] Subramanian A., Tamayo P., Mootha V.K., Mukherjee S., Ebert B.L., Gillette M.A., Paulovich A., Pomeroy S.L., Golub T.R., Lander E.S. (2005). Gene set enrichment analysis: A knowledge-based approach for interpreting genome-wide expression profiles. Proc. Natl. Acad. Sci. USA.

[42] Kim S., Chen J., Cheng T., Gindulyte A., He J., He S., Li Q., Shoemaker B.A., Thiessen P.A., Yu B. (2019). PubChem 2019 update: Improved access to chemical data. Nucleic Acids Res..

[43] Wishart D.S., Feunang Y.D., Guo A.C., Lo E.J., Marcu A., Grant J.R., Sajed T., Johnson D., Li C., Sayeeda Z. (2018). DrugBank 5.0: A major update to the DrugBank database for 2018. Nucleic Acids Res..

[44] Walker D.G., Whetzel A.M., Serrano G., Sue L.I., Lue L.F., Beach T.G. (2016). Characterization of RNA isolated from eighteen different human tissues: Results from a rapid human autopsy program. Cell Tissue Bank.

[45] Russell J.A., Walley K.R. (2010). Vasopressin and its immune effects in septic shock. J. Innate Immun..

[46] Lemke G., Rothlin C.V. (2008). Immunobiology of the TAM receptors. Nat. Rev. Immunol..

[47] Dalrymple N.A., Cimica V., Mackow E.R. (2015). Dengue Virus NS Proteins Inhibit RIG-I/MAVS Signaling by Blocking TBK1/IRF3 Phosphorylation: Dengue Virus Serotype 1 NS4A Is a Unique Interferon-Regulating Virulence Determinant. MBio.

[48] Mishra R., Lahon A., Banerjea A.C. (2020). Dengue Virus Degrades USP33-ATF3 Axis via Extracellular Vesicles to Activate Human Microglial Cells. J. Immunol..

[49] Hai T., Wolford C.C., Chang Y.S. (2010). ATF3, a hub of the cellular adaptive-response network, in the pathogenesis of diseases: Is modulation of inflammation a unifying component?. Gene. Expr..

[50] Phoolcharoen W., Smith D.R. (2004). Internalization of the dengue virus is cell cycle modulated in HepG2, but not Vero cells. J. Med. Virol..

[51] Tokunaga M., Miyamoto Y., Suzuki T., Otani M., Inuki S., Esaki T., Nagao C., Mizuguchi K., Ohno H., Yoneda Y. (2020). Novel anti-flavivirus drugs targeting the nucleolar distribution of core protein. Virology.

[52] Diamond M.S., Zachariah M., Harris E. (2002). Mycophenolic acid inhibits dengue virus infection by preventing replication of viral RNA. Virology.

[53] He J.C., Lu T.C., Fleet M., Sunamoto M., Husain M., Fang W., Neves S., Chen Y., Shankland S., Iyengar R. (2007). Retinoic acid inhibits HIV-1-induced podocyte proliferation through the cAMP pathway. J. Am. Soc. Nephrol..

[54] Machado C.R., Machado E.S., Rohloff R.D., Azevedo M., Campos D.P., de Oliveira R.B., Brasil P. (2013). Is pregnancy associated with severe dengue? A review of data from the Rio de Janeiro surveillance information system. PLoS Negl. Trop. Dis..

[55] Srisutthisamphan K., Jirakanwisal K., Ramphan S., Tongluan N., Kuadkitkan A., Smith D.R. (2018). Hsp90 interacts with multiple dengue virus 2 proteins. Sci. Rep..

[56] Hughes T.R., Marton M.J., Jones A.R., Roberts C.J., Stoughton R., Armour C.D., Bennett H.A., Coffey E., Dai H., He Y.D. (2000). Functional discovery via a compendium of expression profiles. Cell.

[57] Ferreira P.G., Muñoz-Aguirre M., Reverter F., CP S.G., Sousa A., Amadoz A., Sodaei R., Hidalgo M.R., Pervouchine D., Carbonell-Caballero J. (2018). The effects of death and post-mortem cold ischemia on human tissue transcriptomes. Nat. Commun..

[58] Cheng Y.L., Lin Y.S., Chen C.L., Wan S.W., Ou Y.D., Yu C.Y., Tsai T.T., Tseng P.C., Lin C.F. (2015). Dengue Virus Infection Causes the Activation of Distinct NF-κB Pathways for Inducible Nitric Oxide Synthase and TNF-α Expression in RAW264.7 Cells. Mediat. Inflamm..

[59] Barrows N.J., Campos R.K., Powell S.T., Prasanth K.R., Schott-Lerner G., Soto-Acosta R., Galarza-Muñoz G., McGrath E.L., Urrabaz-Garza R., Gao J. (2016). A Screen of FDA-Approved Drugs for Inhibitors of Zika Virus Infection. Cell Host Microbe.

[60] Mazzon M., Ortega-Prieto A.M., Imrie D., Luft C., Hess L., Czieso S., Grove J., Skelton J.K., Farleigh L., Bugert J.J. (2019). Identification of Broad-Spectrum Antiviral Compounds by Targeting Viral Entry. Viruses.

[61] Ko H.C., Wang Y.H., Liou K.T., Chen C.M., Chen C.H., Wang W.Y., Chang S., Hou Y.C., Chen K.T., Chen C.F. (2007). Anti-inflammatory effects and mechanisms of the ethanol extract of Evodia rutaecarpa and its bioactive components on neutrophils and microglial cells. Eur. J. Pharmacol..

[62] Dai J.P., Li W.Z., Zhao X.F., Wang G.F., Yang J.C., Zhang L., Chen X.X., Xu Y.X., Li K.S. (2012). A drug screening method based on the autophagy pathway and studies of the mechanism of evodiamine against influenza A virus. PLoS ONE.

[63] Pollard B.S., JC B.L., Pollard J.R. (2020). Classical Drug Digitoxin Inhibits Influenza Cytokine Storm, With Implications for Covid-19 Therapy. In Vivo.

[64] Smith J.L., Stein D.A., Shum D., Fischer M.A., Radu C., Bhinder B., Djaballah H., Nelson J.A., Früh K., Hirsch A.J. (2014). Inhibition of dengue virus replication by a class of small-molecule compounds that antagonize dopamine receptor d4 and downstream mitogen-activated protein kinase signaling. J. Virol..

[65] Wang P., Wang Z., Liu J. (2020). Role of HDACs in normal and malignant hematopoiesis. Mol. Cancer.

[66] Delgado F.G., Cárdenas P., Castellanos J.E. (2018). Valproic Acid Downregulates Cytokine Expression in Human Macrophages Infected with Dengue Virus. Diseases.

[67] Choy M.M., Zhang S.L., Costa V.V., Tan H.C., Horrevorts S., Ooi E.E. (2015). Proteasome Inhibition Suppresses Dengue Virus Egress in Antibody Dependent Infection. PLoS Negl. Trop. Dis..

[68] Mukhopadhyay P., Rajesh M., Cao Z., Horváth B., Park O., Wang H., Erdelyi K., Holovac E., Wang Y., Liaudet L. (2014). Poly (ADP-ribose) polymerase-1 is a key mediator of liver inflammation and fibrosis. Hepatology.

