# Supplementary material for: Multi-Tissue Transcriptomic-Informed In Silico Investigation of Drugs for the Treatment of Dengue Fever Disease
Source: Viruses. 2021 Aug 4;13(8):1540. doi: 10.3390/v13081540 (PMC8402662; doi:10.3390/v13081540)
Supplement: Supplementary file 1 [file viruses-13-01540-s001.zip › Supplementary data/PaperDengueCuba_transcriptomics_Supplements_Last version.pdf]

Multi-tissue transcriptomic-informed *in silico* investigation of drugs for the treatment of dengue fever disease

Supplementary file

Table S1- Additional information on the individuals from whom samples were collected for transcriptomics.

Group	ID number	Date of symptom onset	Date of death	Age	Gender	Results of dengue RT-PCR (2018) and dengue nested RT-PCR (2014) n tissues	Province	Clinical classification
Cases	37478	26 July 2018	31 July 2018	71	F	DENV-2 in spleen and liver	Camagüey	DSS
	39539	24 July 2018	28 July 2018	49	F	DENV-2 in spleen and liver	Camagüey	DSS
	39538	22 July 2018	30 July 2018	59	M	DENV-2 in liver	Camagüey	DSS
	900	7 October 2014	13 October 2014	66	F	DENV-2 in liver	Habana	DSS
	875	21 October 2014	26 October 2014	48	M	DENV-2 in liver	Habana	DSS
Controls	F1	NA	July 2018	32	M	Negative	Habana	NA
	F2	NA	July 2018	24	F	Negative	Habana	NA
	F4	NA	July 2018	47	M	Negative	Habana	NA

DWWS – dengue with warning signs.

NA- not applicable.

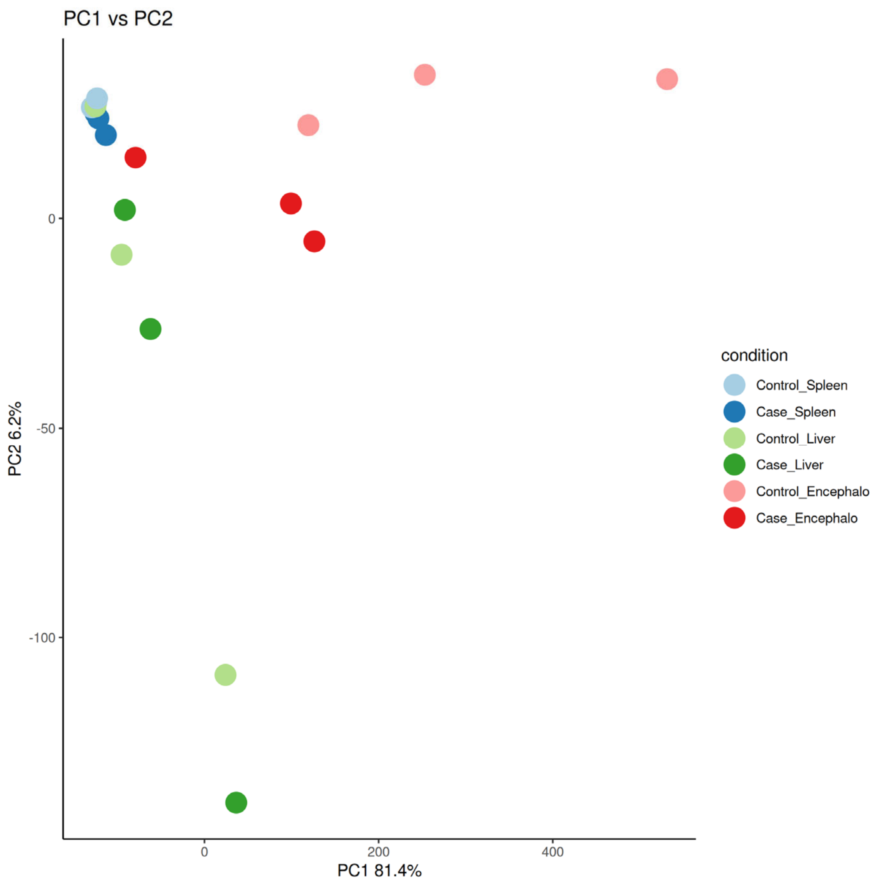

Figure S1- PCA plot (PC1 explaining 81.4% of variation vs. PC2 explaining 6.2% of variation) of the human transcriptomic profile of the spleen, liver and encephalon tissues.

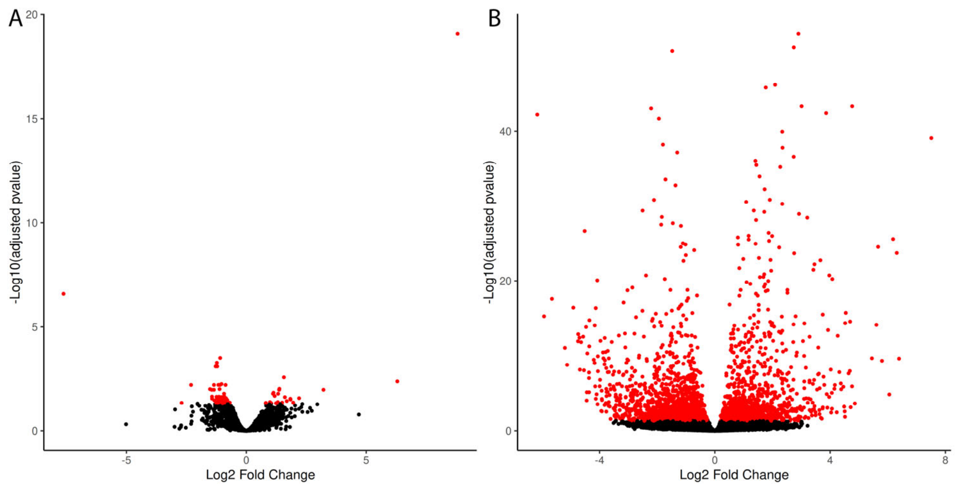

Figure S2- Volcano plots for the gene differential expression in the (A) spleen and (B) liver cell datasets. The points represented on red are the statistically significant genes. The negative side of the graph refers to downregulated genes in patients when compared to controls; the positive side of the graph refers to upregulated genes in patients when compared to controls.

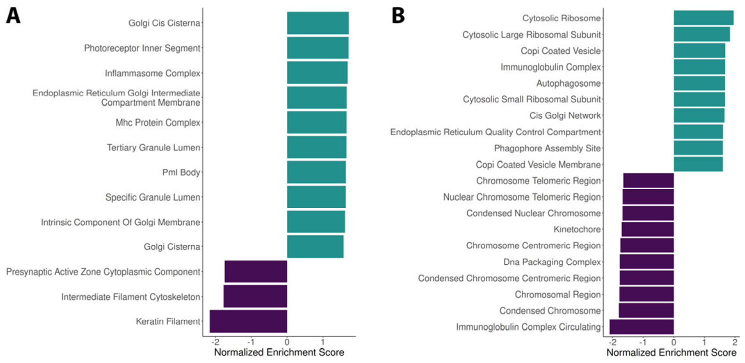

Figure S3- Top 20 Gene Ontology Cellular Component (GO-CC) pathways in the (A) Spleen and (B) Liver cells (cut-off value; nominal p-value < 0.05 and FDR < 0.25). Positive NES (in purple) show upregulated pathways in the infected individuals versus controls, while negative NES values (in green) mean downregulated pathways in the infected individuals versus controls.

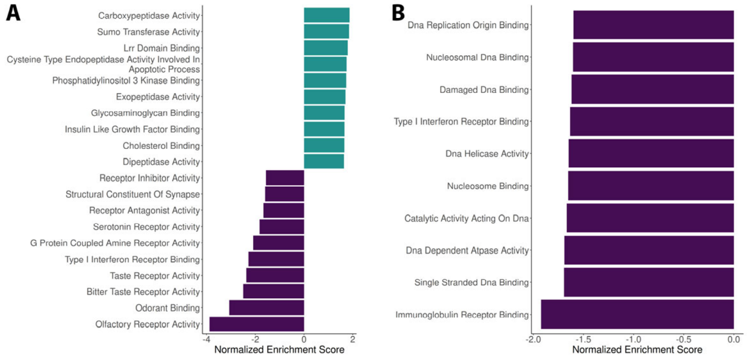

Figure S4- Top 20 Gene Ontology Molecular Function (GO-MF) pathways in the (A) Spleen and (B) Liver cells (cut-off value; nominal p-value < 0.05 and FDR < 0.25). Positive NES (in purple) show upregulated pathways in the infected individuals versus controls, while negative NES values (in green) mean downregulated pathways in the infected individuals versus controls.

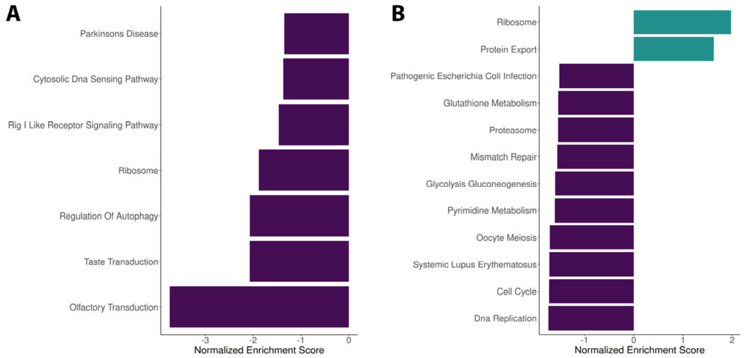

Figure S5- Top 20 significantly enriched KEGG pathways in the (A) Spleen and (B) Liver cells (cut-off value; nominal p-value < 0.05 and FDR < 0.25). Positive NES (in purple) show upregulated pathways in the infected individuals versus controls, while negative NES values (in green) mean downregulated pathways in the infected individuals versus controls.

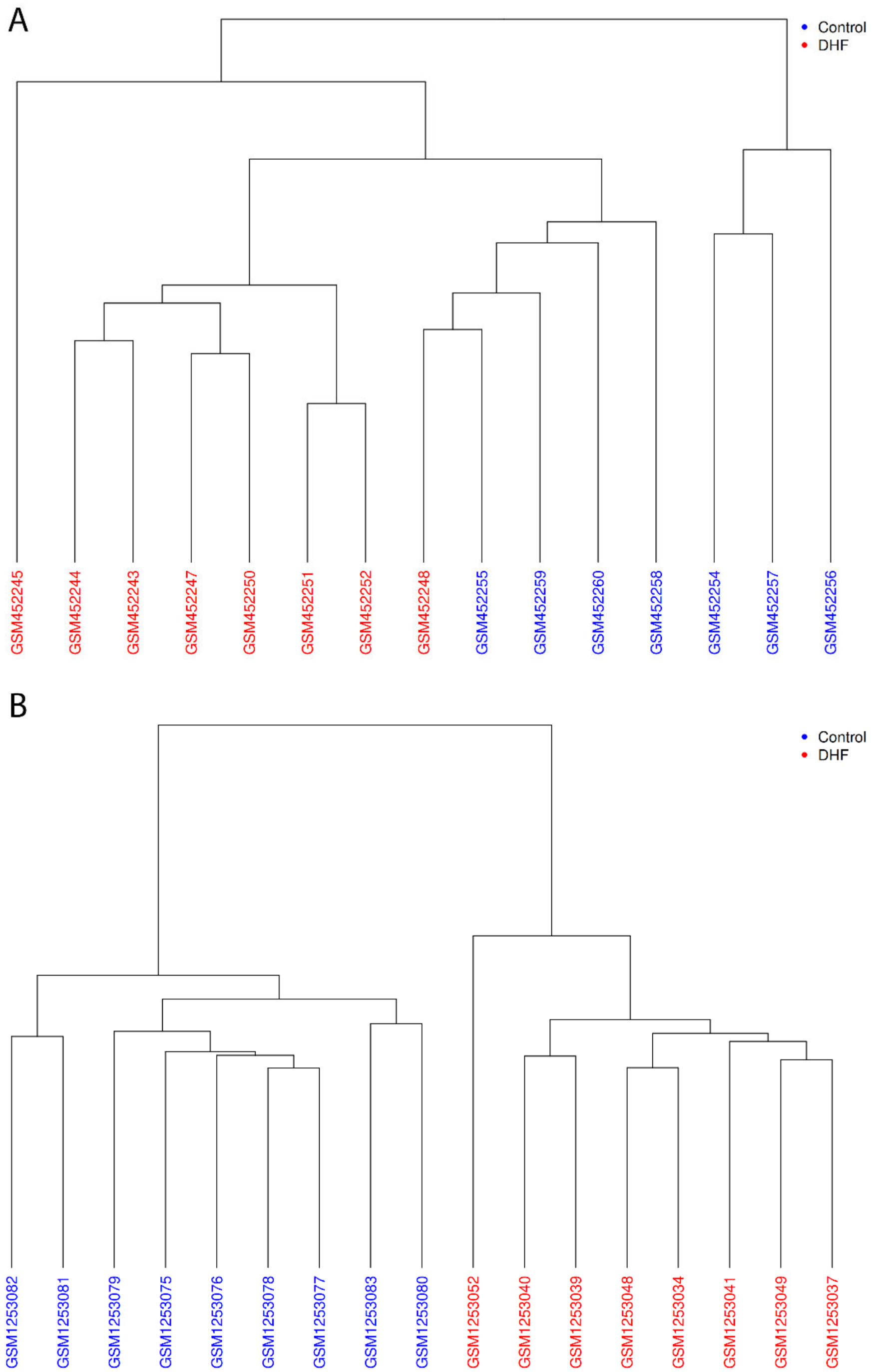

Figure S6- Dendrograms for the gene expression profiles in the two blood datasets: (A) GSE18090 and (B) GSE51808. The red points indicate the infected dengue haemorrhagic fever individuals (DHF), while the blue points indicate control samples.

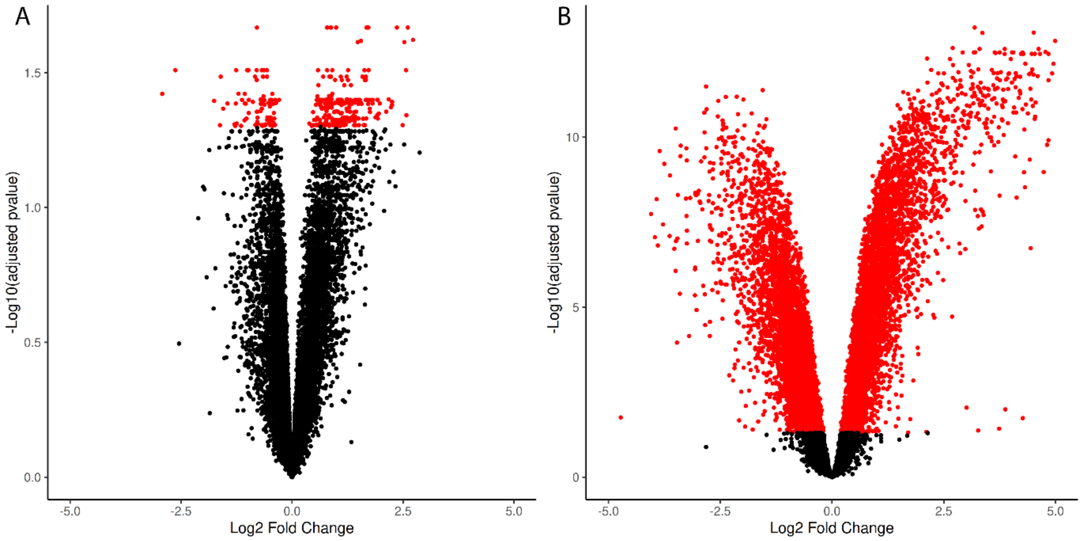

Figure S7- Volcano plots for the gene differential expression in the two blood datasets: (A) GSE18090 and (B) GSE51808. The points represented in red are the statistically significant genes. The negative side of the graph refers to downregulated genes in patients when compared to controls; the positive side of the graph refers to upregulated genes in patients when compared to controls.

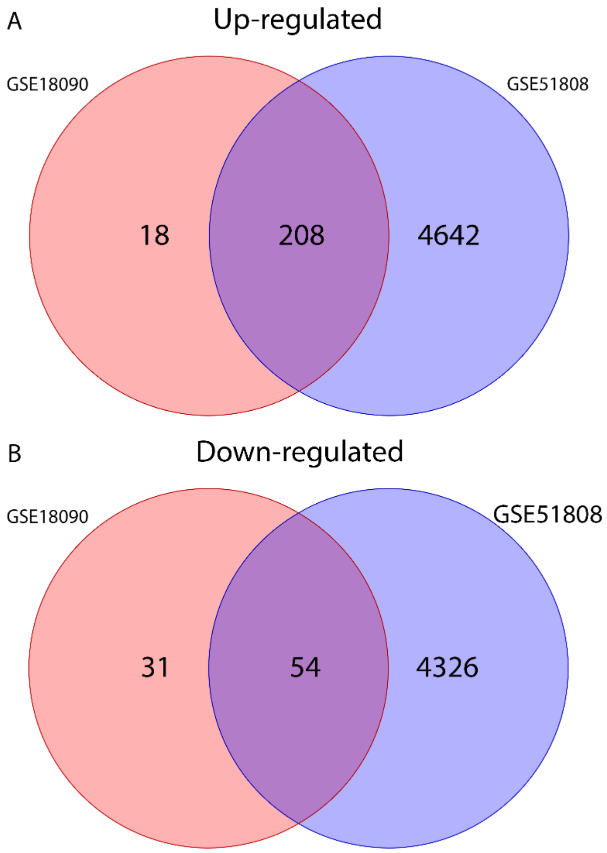

Figure S8- Venn diagrams of the upregulated (A) and downregulated (B) genes in the two blood datasets.

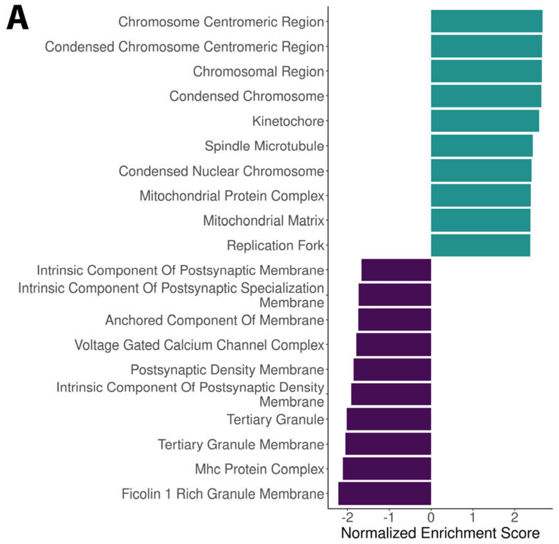

Figure S9- Top 20 Gene Ontology Cellular Component (GO-CC) pathways in the (A) GSE18090 and (B) GSE51808 (cut-off value; nominal p-value < 0.05 and FDR < 0.25). Positive NES (in purple) show upregulated pathways in the infected individuals versus controls, while negative NES values (in green) mean downregulated pathways in the infected individuals versus controls.

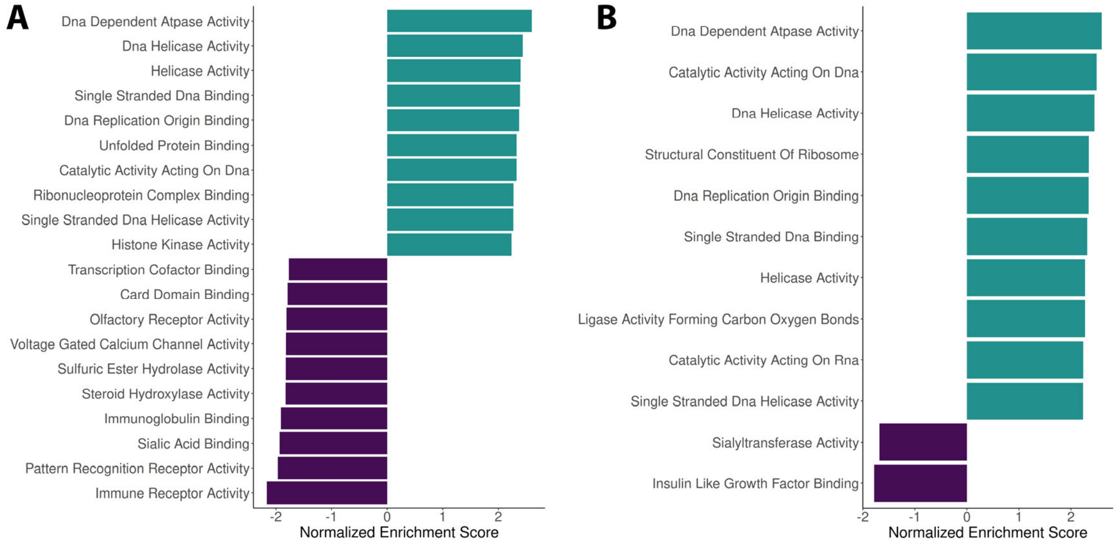

Figure S10- Top 20 Gene Ontology Molecular Function (GO-MF) pathways in the (A) GSE18090 and (B) GSE51808 (cut-off value; nominal p-value < 0.05 and FDR < 0.25). Positive NES (in purple) show upregulated pathways in the infected individuals versus controls, while negative NES values (in green) mean downregulated pathways in the infected individuals versus controls.

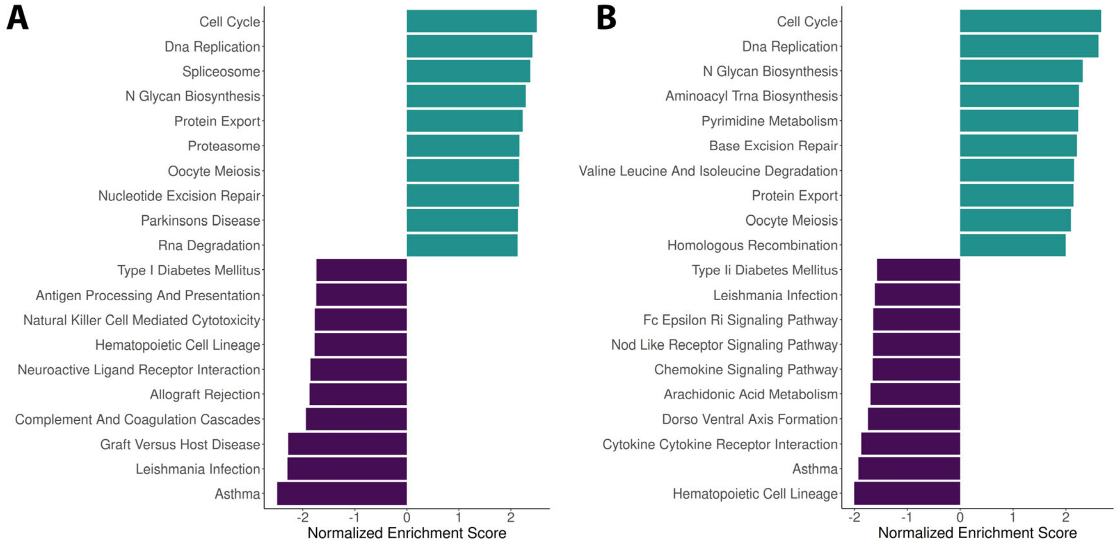

Figure S11- Top 20 significantly enriched KEGG pathways in the (A) GSE18090 and (B) GSE51808 (cut-off value; nominal p-value < 0.05 and FDR < 0.25). Positive NES (in purple) show upregulated pathways in the infected individuals versus controls, while negative NES values (in green) mean downregulated pathways in the infected individuals versus controls.
